# Mitochondrial Genome Architecture and Evolutionary Origin of the Yao Silkworm, a Living Fossil of the Domestic Silkworm *Bombyx mori* (Lepidoptera: Bombycidae)

**DOI:** 10.1093/jisesa/ieac014

**Published:** 2022-03-18

**Authors:** Gui-Zheng Zhang, Yu-Li Zhang, Wei Wei, Yu-Ping Li, Yan-Qun Liu, Li-Hui Bi, Cheng Lu

**Affiliations:** 1 Guangxi Institute of Sericulture Science, 10 Xiajun Road, Nanning 530007, China; 2 Department of Sericulture, Shenyang Agricultural University, 120 Dongling Road, Shenyang 110866, China; 3 State Key Laboratory of Silkworm Genome Biology, Southwest University, 2 Tiansheng Road, Chongqing 400715, China

**Keywords:** *Bombyx mori*, Yao silkworm, sequence comparison, variation pattern, phylogenetic relationship

## Abstract

The Yao silkworm is a unique silkworm resource producing yellow flat plate silk that has only been reared by the Baiku Yao ethnic group in Nandan County, Guangxi Province, China for a thousand years. Here, we report the mitochondrial genomes (mitogenomes) of five Yao silkworm strains and 10 local Guangxi strains of the domestic silkworm (*Bombyx mori*) L. (Lepidoptera: Bombycidae), and use the resulting mitogenomes and the available *Bombyx* mitogenomes to characterize their genome architecture and trace the evolutionary origin of the Yao silkworm. The five Yao silkworm mitogenomes exhibited genome architectures identical to typical set of 37 mitochondrial genes (13 protein-coding genes, 22 transfer RNAs, and two ribosomal RNAs) and a high level of genome sequence similarity with the domestic silkworm. Mitogenome-based phylogenetic reconstruction provided solid evidence that the Yao silkworm shares a common ancestor with the domestic silkworm. Sliding window analysis uncovered a distinct variation pattern in the mitogenome between the Yao silkworm and the other domestic silkworm strains. The phylogenetic analyses revealed a basal placement of the Yao silkworm among all available domestic silkworm strains, indicating that the Yao silkworm is an ancient population of the domestic silkworm. Our data indicated that the Yao silkworm (*B. mori*) is a lineage of the domestic silkworm, which for the first time provides insights into the origin of the Yao silkworm.

The Baiku Yao ethnic group in Nandan County, Guangxi Province, China has been regarded as a ‘living fossil of human civilization’ by the United Nations Educational, Scientific and Cultural Organization (Tan and Jiang 2012, Guan et al. 2014). Baiku Yao, a branch of the Yao ethnic group with a population size of approximately 30,000, is so named because the men wear white knee-length trousers and scarves, while the women wear pleated skirts and black scarves (Wen 2006). Since directly entering modern society from a primitive society, the Baiku Yao ethnic group still carries much cultural information on the transition between a matrilineal society and a patrilineal society. Because the Baiku Yao ethnic group seldom interacts with other nationalities, their unique customs and cultures such as clothing, intraethnic marriages, funerals, bronze drums, and dietary habits are still completely preserved (Yan et al. 2012). 

The present study focused on the Yao silkworm, which has just been known by silkworm researchers since 1986. The Yao silkworm is reared only in 29 villages in Nandan County (N 24°25′ ~ 25°37′; E 107°01′ ~ 107°53′) (Fig. 1A) and has been reared to produce flat plate silk by the Baiku Yao ethnic group (Fig. 1B) for a thousand years; thus, it has been regarded as a ‘living fossil of Chinese silk culture’ (Lu et al. 2013). Similar to its ethnic culture, the Yao silkworm is a highly pure local population (Guan et al. 2014). The legend has it that the Yao silkworm was granted by the mountain deities Yaojia and Lanla from its wild counterpart, which suggests that the Yao silkworm might have its own origin history (Lu et al. 2013) that is different from the domestic silkworm (*Bombyx mori*) L. (Lepidoptera: Bombycidae), whose birthplace might be in the Yellow River basin (Jiang 1982, Luo 1993). Generally, the Yao silkworm eggs are incubated by women who carry them close to their skin to hasten their hatching. The larvae are reared at home for 40–50 d, and the mature larvae are placed on a board to produce flat plate silk that can then be used for clothing, weddings, and funerals (Tan and Jiang 2012). The Yao silkworm is a unique silkworm resource characterized by the production of univoltine, flat plate silk and natural golden yellow silk, and is a natural trimolter (Guan et al. 2014, Bai et al. 2015); however, genetic information about the Yao silkworm remains severely limited.

**Fig. 1.**
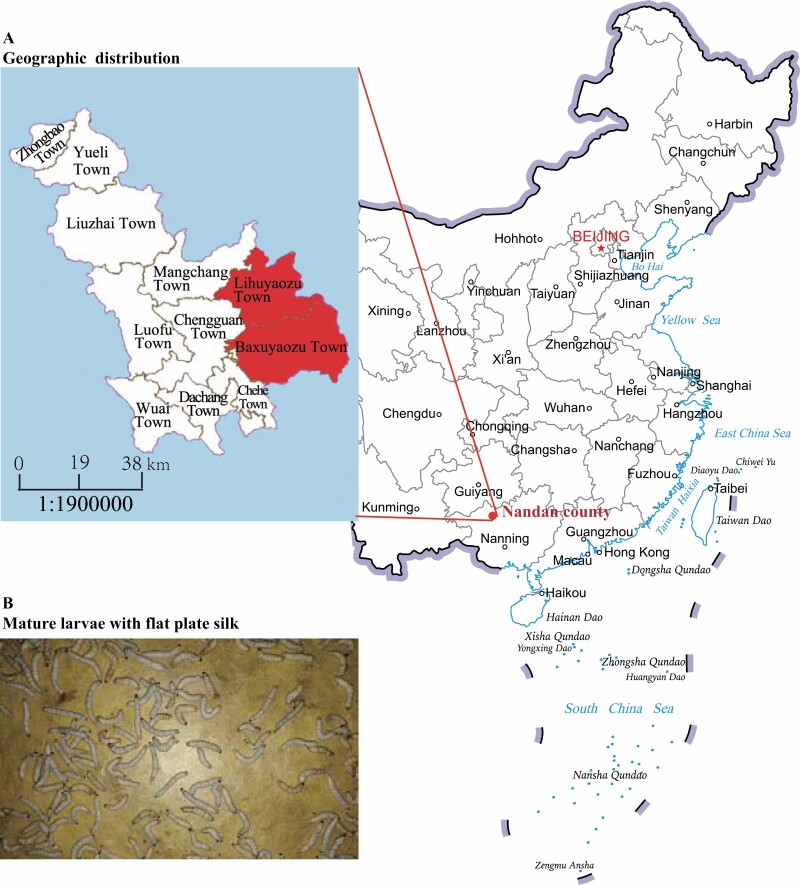
Geographic distribution of the Yao silkworm (Nandan County, Guangxi Province, China). (A) The main distribution area of the Yao silkworm is highlighted in red. (B) The mature larvae and yellow flat plate silk are shown.

Chinese has utilized domestic silkworms for over 5000 years (Goldsmith et al. 2005, Xiang et al. 2005). Historical and archaeological evidence has suggested that the domestication of domestic silkworms might have occurred independently at multiple centers, including the Yellow River basin, the Yangtse River basin, and ethnic minority areas (Jiang 1982). In the past decade, enormous efforts on understanding the origin of the domestic silkworm have been made at the molecular level and have confirmed that the wild silkworm Chinese *Bombyx mandarina* Moore (Lepidoptera: Bombycidae) is the wild ancestor of the domestic silkworm, instead of the Japanese *B. mandarina* (Lu et al. 2002, Li et al. 2005, Xiang et al. 2005, Arunkumar et al. 2006, Li et al. 2010). Genome resequencing of a large number of the domestic silkworm strains has also suggested that the domestic silkworm might be involved in a short domestication event (Xia et al. 2009) and that silkworms might have been initially domesticated as trimolting lines (Xiang et al. 2018). The fact that wild silkworms (*B. mandarina*) are distributed throughout Asia and exhibit great diversity (Astaurov and Rovinskaya 1959) makes this issue more complex. A recent study of silkworms revealed that Chinese *B. mandarina* populations can be divided into two genetically distinctive subtypes, northern Chinese *B. mandarina* and southern Chinese *B. mandarina*, and that the former is the wild ancestor of the domestic silkworm (Chen et al. 2019). The Yao silkworm is a case in point (Luo et al. 1993, Lu et al. 2013); however, it was not included in these previous studies.

Does the Yao silkworm have its own origin history? The fact that no wild counterparts have been recorded in Guangxi opposes the theory of the independent domestication event of the Yao silkworm. In this context, we undertook the present work on the origin of the Yao silkworm. Our primary objective was to explore the evolutionary origin of the Yao silkworm through mitochondrial genome (mitogenome) comparison. The animal mitogenome encodes a typical set of 37 mitochondrial genes, including 13 protein-coding genes (PCGs), 22 transfer RNA (tRNA) genes, two ribosomal RNA (rRNA) genes, and a noncoding A+T-rich region. Due to maternal inheritance, small genome size, and relatively high evolutionary rate, the animal mitogenome has played an increasingly important role in phylogenetic studies (Cameron 2014). A recent study has suggested that the whole mitogenome will provide good access to the maternal origin of the domestic silkworm (Chen et al. 2019).

In the present study, complete mitogenomes from five strains of the Yao silkworm and 10 local Guangxi strains of the domestic silkworm were determined. By combining our new data with previously reported sequences, we obtained a dataset of 71 mitogenome sequences for all the available *Bombyx* data. Then, these sequences were compared to characterize their genome architecture, and mitogenome-based phylogenetic analyses were performed to trace the origin of the Yao silkworm.

## Materials and Methods

### Silkworm Collection

In the present study, five inbred strains of the Yao silkworm and 10 local Guangxi strains of the domestic silkworm were used (Table 1). All these strains were preserved normally at the Guangxi Institute of Sericulture Science, China or the Silkworm Gene Bank of Southwest University, China. The five Yao silkworm strains exhibiting different agronomic characteristics were initially collected from Nandan County, Guangxi, China.

**Table 1.** The detailed information of the mitogenomes determined in this study

Species/strain ID	Source	GenBank no.	Genome size/bp
Yao silkworm			
Yao_2B1	Guangxi, China	MW158377	15,656
Yao_2B2	Guangxi, China	MW158378	15,657
Yao_17W	Guangxi, China	MN027269	15,656
Yao_17B	Guangxi, China	MW158380	15,656
Yao_2W	Guangxi, China	MW158379	15,656
*Bombyx mori*			
Bm_NC9R	Guangxi, China	MW158384	15,656
Bm_97	Guangxi, China	MW158372	15,656
Bm_932	Guangxi, China	MW158374	15,656
Bm_35	Guangxi, China	MW158369	15,653
Bm_76	Guangxi, China	MW158370	15,653
Bm_P2286	Guangxi, China	MW158385	15,656
Bm_750Y7A	Guangxi, China	MW158373	15,656
Bm_Xianghui	Guangxi, China	MW158386	15,656
Bm_7532	Guangxi, China	MW158375	15,656
Bm_Furong	Guangxi, China	MW158382	15,656

### Mitogenome Sequencing, Assembly and Annotation

A single pupa of each strain was used to extract the total genomic DNA using a Wizard Genomic DNA Purification Kit (Promega, Madison, WI). Standard Illumina sequencing was performed in the paired-end mode by Novogene Biotech Co., Ltd. (Beijing, China). We generated ~15× coverage for each sequenced sample. The raw short read data of 150 bp were then used to assemble the mitogenome, with strain C108 of the domestic silkworm and its annotation (AB070264; Yukuhiro et al. 2002) as the reference. Raw short reads were mapped onto the reference mitogenome by program SOAP v1.09 (Li et al. 2008). SOAPsnp (Li et al. 2009) was used to calculate the posterior probability of each possible genotype at every genome position from the alignment results. The consensus sequence was then structured based on the highest probability. New mitogenome sequences were annotated in MITOS (Bernt et al. 2013) and then refined manually by aligning against the reference sequence.

### Data Collection and Sequence Comparison

We downloaded 38 other mitogenome sequences of the domestic silkworm available to date from the National Centre for Biotechnology Information. By combining our new data with previously reported sequences, we generated a dataset of 71 whole mitogenome sequences of the domestic silkworm and its wild relatives. Nucleotide sequences were aligned by using Clustal Omega with the default settings (https://www.ebi.ac.uk/Tools/msa/clustalo/) (Sievers et al. 2011) and revised by eye. DnaSP v6 (Rozas et al. 2017) was used for sliding window analysis to determine the nucleotide diversity of the mitogenomes of silkworms, with a step size of 200 bp and a window length of 800 bp.

### Phylogenetic Reconstruction

The entire mitogenomes of the Yao silkworm and the domestic silkworm available were used to infer their phylogenetic relationships with maximum likelihood analysis and Bayesian inference algorithm. Two non-*Bombyx* species, *Antheraea pernyi* Guérin-Méneville (Lepidoptera: Saturniidae) (GenBank Accession no. AY242996), belonging to Saturniidae and *Rondotia menciana* Moore (Lepidoptera: Bombycidae) (Kong and Yang 2015), belonging to Bombycidae served as the outgroups. The GTR+G+I model was chosen according to the lowest Bayesian Information Criterion scores with MEGA X (Kumar et al. 2018). Maximum likelihood analysis was conducted with IQ-TREE 1.6.12 (Nguyen et al. 2015), and the bootstrap values were evaluated with 1,000 iterations. Bayesian inference analysis was performed using MrBayes v3.1.2 (Huelsenbeck and Ronquist 2001). The posterior distributions were estimated with Markov Chain Monte Carlo (MCMC) sampling. In this study, the MCMC search was conducted for 11,500,000 generations, with sampling every 100 generations, and the average standard deviation of split frequencies reached 0.0076. The first 25% of trees were then discarded as ‘burn-in’. Two independent runs were performed for each method. The Interactive Tree Of Life (https://itol.embl.de) was used to visualize the obtained phylogenetic trees (Letunic and Bork 2021).

## Results

### Characterization of the Mitogenome of the Yao Silkworm

In this study, the mitogenomes of five strains of the Yao silkworm and 10 local Guangxi strains of the domestic silkworm were first determined to characterize their genome architecture. These five Yao silkworm strains exhibited a low cocoon shell ratio of approximately 7.8%, which was comparable to the wild silkworm *B. mandarina* (7–8%), but smaller than that of the domestic silkworm *B. mori* (15%; Li et al. 2000). All 15 mitogenomes presented here were double-stranded circular molecules and contained 37 typical mitochondrial genes (13 PCGs, 22 tRNAs, and 2 rRNAs) and an A+T-rich region, with identical genome architectures, gene orientation, and gene order. The mitogenome of strain Yao_2B2 was 15,657 bp in size, while that of the other four strains (2B1, 17W, 17B, and 2W) was 15,656 bp. The genome size of the Yao silkworm was well within the range detected in the sequenced *B. mori* strains but shorter than those found in *B. mandarina*. The composition of the five Yao silkworm mitogenomes was highly biased toward the A+T nucleotide (81.4%), which was in line with that of the sequenced *B. mori* strains. In the mitogenomes of the Yao silkworm, the 12 PCGs started with ATN (ATG/ATA/ATT), while *COI* started with CGA. Ten PCGs ended with TAA, while *COI*, *COII*, and *NAD4L* ended with the incomplete stop codons T or TA. Twenty-two typical tRNA genes were identified in each of the mitogenomes of the Yao silkworm, with sizes ranging between 65 bp and 73 bp. All tRNAs in each of the mitogenomes of the Yao silkworm, except for *tRNA*^*Ser*^*(AGN),* which lacked a stable dihydrouridine arm, showed the typical cloverleaf secondary structure observed in the mitochondrial tRNA genes of previously reported mitogenomes of silkworms.

The A+T-rich region in strain Yao_2B2 was 495 bp, while those of the other four strains were 494 bp. The size of the A+T-rich region of the Yao silkworm was highly similar to those observed in the sequenced *B. mori* strains. A 126 bp repeat element has been well characterized in *Bombyx* species (Yukuhiro et al. 2002). Only one copy of this repeat element was found in each of the mitogenomes of the five Yao silkworm strains, as has been observed in 48 sequenced *B. mori* strains and 15 Chinese *B. mandarina* collections (Pan et al. 2008, Chen et al. 2019), whereas three copies of the tandem replication of this repeat element have been observed in the Japanese *B. mandarina* (Yukuhiro et al. 2002). The length differences in silkworm mitogenomes are primarily the result of the size variation in the A+T-rich region (Pan et al. 2008).

**Fig. 2.**
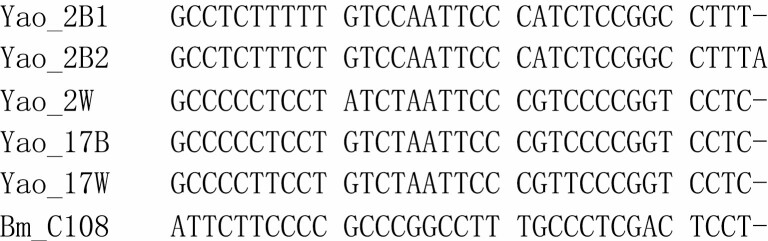
Variable sites in the mitogenomes of five Yao silkworm strains (2B1, 2B2, 2W, 17B, and 17W) and the domestic silkworm strain (C108-AB070264). In total, 35 variable sites were identified among these six strains.

Among these five mitogenome sequences, we identified 13 single nucleotide variants (SNVs) across the 15,657 bp consensus aligned sequence (Fig. 2), thus indicating that they were very closely related, consistent with their recent selection history (Zhang et al. 2017). Twelve of 13 SNVs occurred in the coding region, and one occurred in the A+T-rich region. Only one variable site was found between Yao_2W and Yao_17B, while 11 variant sites occurred between Yao_2W and Yao_2B1. When compared to the first reference mitogenome sequence of *B. mori* strain C108 (AB070264), the number of variant sites between each pair ranged from 23 (Yao_17B and Yao_17W) to 25 (Yao_2B1 and Yao_2B2).

### The Yao Silkworm Has Highly Sequence Similarity with the Domestic Silkworm, but Exhibits a Different Variation Pattern

Within the *Bombyx* silkworms, four distinct haplotype clades have been identified based on the mitogenome sequence, including the *B. mori* clade, Japanese *B. mandarina* clade, northern Chinese *B. mandarina* clade, and southern Chinese *B. mandarina* clade (Chen et al. 2019). The five mitogenome sequences of the Yao silkworm, together with 10 mitogenome sequences of local Guangxi strains of the domestic silkworm determined in this study, were aligned with 38 known mitogenome sequences of the domestic silkworm, and the result indicated a high similarity of the nucleotide sequences between the Yao silkworm and *B. mori* (data not shown). Analysis of the genetic distance also indicated that the Yao silkworm had the smallest genetic distance from strains of the *B. mori* clade, a value of 0.002 comparable to that within strains of the domestic silkworm (Table 2). Both sequence alignment and genetic distance showed that the Yao silkworm is a lineage of the *B. mori* clade rather than the *B. mandarina* clade.

**Table 2.** Pairwise Kimura-2-parameter genetic distance within or between group of silkworms

		1	2	3	4	5
1	Yao silkworm (*n* = 5)	0.001				
2	*B. mori* (*n* = 48)	0.002	0.002			
3	Northern Chinese *Bombyx mandarina* (*n* = 3)	0.007	0.008	0.004		
4	Southern Chinese *Bombyx mandarina* (*n* = 13)	0.023	0.023	0.023	0.005	
5	Japanese *Bombyx mandarina* (*n* = 2)	0.034	0.035	0.034	0.025	0.003

**Fig. 3.**
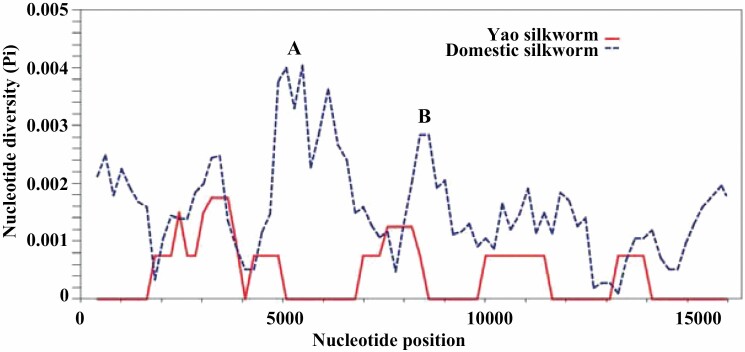
Sliding window analysis of the mitogenomes of the Yao silkworm (*n* = 5) and the domestic silkworm (*n* = 48). Window length: 800 bp; step size: 200 bp. X-axis: position of the middle point of the window. Y-axis: nucleotide diversity per window. Region A is located between *COIII* and *tRNA*^*Phe*^, and Region B is positioned between *tRNA*^*His*^ and *tRNA*^*Thr*^.

Sliding window analysis was conducted to investigate the difference in variation patterns between the Yao silkworm and the domestic silkworm strains. The average value of nucleotide diversity (Pi) in the five Yao silkworm strains was 0.00042, while that of the 48 domestic silkworm strains was 0.00158. Through the sliding analysis, we found two regions with distinct variation patterns between the two groups (Fig. 3). One was the region between *COIII* and *tRNA*^*Phe*^, a fragment of approximately 2.3 kb. Within this region, the Pi values of 3 mutation hotspots for the 48 domestic silkworm strains were significantly higher (>0.003). The other region, a fragment of approximately 1.8 kb, was positioned between *tRNA*^*His*^ and *tRNA*^*Thr*^: one mutation hotspot for the 48 domestic silkworm strains was found with a Pi value of 0.0028 (*tRNA*^*His*^-*ND4*).

### The Yao Silkworm Shares a Common Ancestor with the Domestic Silkworm, but Possesses a Basal Position in the Phylogenetic Tree

**Fig. 4.**
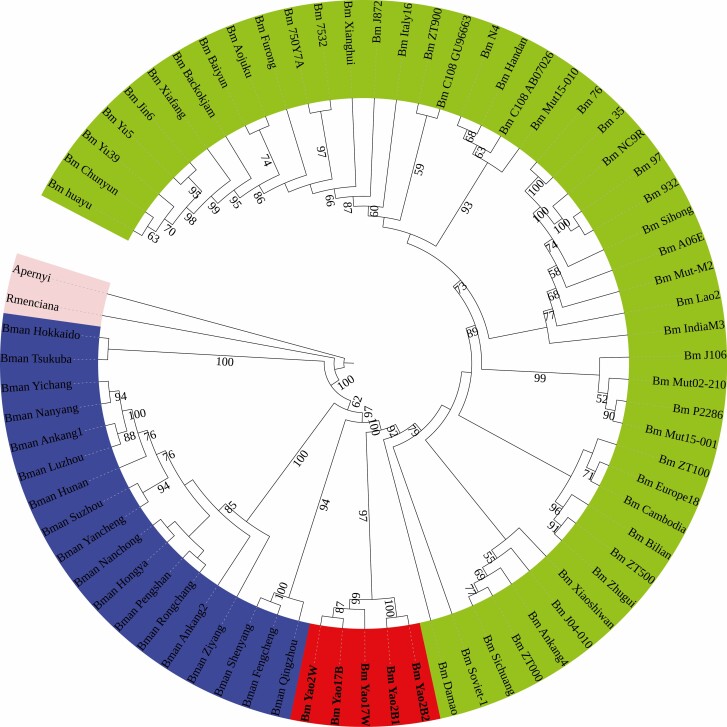
Silkworm mitochondrial phylogeny built by the maximum likelihood method with the GTR + G + I model based on the nucleotide sequence of whole mitogenomes of *Bombyx*. The numbers close to the nodes specify bootstrap values. Except for the outgroups *Antheraea pernyi* and *Rondotia menciana*, each *Bombyx* sample is represented by the strain name. The topology based on Bayesian inference that exhibits a highly similar phylogenetic relationship with the maximum likelihood method is not shown.

To evaluate the phylogenetic relationship of the Yao silkworm and the other domestic silkworm strains, we performed a breakthrough phylogenetic analysis based on 71 whole mitogenome sequences of *Bombyx* silkworms, including five Yao silkworm sequences, 48 domestic silkworm sequences, three northern Chinese wild silkworm sequences, 13 southern Chinese wild silkworm sequences, and two Japanese wild silkworm sequences. The mitogenome-based phylogenetic tree provided us with a well-resolved phylogeny of *Bombyx* silkworms (Fig. 4). In the phylogenetic tree, the five Yao silkworm strains and 48 domestic silkworm strains clustered together with a great value support of 100%, suggesting that they share a common ancestor; that is, the Yao silkworm is a lineage of the domestic silkworm. The fact that the Yao silkworm and 48 domestic silkworm strains collected from all over the world formed a monophyletic group further provided molecular evidence for a single-domestication origin of the domestic silkworm. Accordingly, the phylogenetic tree implied that the legendary early independent domestication event might not have occurred in the Yao silkworm, which is in good agreement with the fact that no wild silkworms have been recorded in Guangxi, China. Moreover, phylogenetic analyses revealed the basal placement of the Yao silkworm among all available domestic silkworm strains.

## Discussion

In this study, the mitogenomes of five Yao silkworm strains were characterized and then used to trace their evolutionary relationship with the domestic silkworm. The Yao silkworm is a unique silkworm resource only found in 29 villages in Nandan County located in South China that has been utilized to produce silk by the Baiku Yao ethnic group for a thousand years. Due to the high morphological similarity, the Yao silkworm has been suggested as a relatively pure local population of the domestic silkworm (Guan et al. 2014). On the origin of the Yao silkworm, no historical records are available, although the legend has it that the Yao silkworm was developed by the mountain deities (Tan and Jiang 2012, Lu et al. 2013). Some researchers hold that the Yao silkworm might have its own origin history, which is different from the domestic silkworm, in which domestication might have occurred within the area adjacent to the Yellow River (Jiang 1982, Luo 1993). The fact that no wild counterparts have been obtained or recorded in Guangxi Province makes the independent domestication event of the Yao silkworm impossible. Our analysis of the mitogenomes of five Yao silkworm strains revealed a great similarity with the domestic silkworm strains in genome size, gene composition, and genomic organization and a large difference with the wild silkworm *B. mandarina*. Our phylogenetic analysis based on the mitogenome further clustered the Yao silkworm together with the domestic silkworm strains. This is the first study to show molecular evidence that the Yao silkworm shares a common ancestor with the domestic silkworm strains.

Sliding window analysis uncovered a different variation pattern in the mitogenome between the Yao silkworm and the other domestic silkworm strains. Further analysis identified two regions with distinct variation patterns between the two groups: one was located between *COIII* and *tRNA*^*Phe*^, and the other was located between *tRNA*^*His*^ and *tRNA*^*Thr*^. The distinct variation pattern between the two groups may be derived from their different selection pressures or genetic drift. The domestic silkworm was successfully domesticated from the wild silkworm through artificial selection for thousands of years, with improvements in the cocoon shell ratio from 7–8% in the wild silkworm to 15% in the domestic silkworm (Li et al. 2000). Our previous investigation indicated that the Yao silkworm exhibits a low cocoon shell ratio (7.8%), which is in line with the fact that the Yao silkworm is reared in a primitive state (Zhang et al. 2017). The findings from molecular and economic trait analyses suggest that the Yao silkworm is a unique silkworm resource that should be given more attention for its perseveration. In the near future, comparative analysis of the nucleic genomes between the Yao silkworm and the domestic silkworm will provide novel insights into the genetic improvement of the domestic silkworm.

Our mitogenome-based phylogenetic analysis revealed the basal placement of the Yao silkworm within all available domestic silkworm strains. The basal placement of Yao silkworm that exhibits natural trimolter further supports the view that the domestic silkworm might have been initially domesticated as trimoulting lines according to genome resequencing (Li et al. 2010). The findings presented here suggest that the Yao silkworm is a relatively primitive lineage of the domestic silkworm that can be regarded as the living fossil of the domestic silkworm.

In summary, our study presented 15 complete mitogenomes from five Yao silkworm strains and 10 local Guangxi strains of the domestic silkworm. By comparative analysis of 71 complete mitogenomes including our new data and previously reported sequences of *Bombyx*, we found that the Yao silkworm is a lineage of the domestic silkworm despite reproductive isolation for a long period of time between them. We also uncovered a distinct variation pattern in the mitogenome between the Yao silkworm and the other domestic silkworm strains. The basal placement of the Yao silkworm in the phylogenetic tree among all available domestic silkworm strains indicated that the Yao silkworm is an ancient population of the domestic silkworm (*B. mori*) that can be regarded as the living fossil of the domestic silkworm.

## References

[CIT0001] Arunkumar, K. P., M. Metta, and J. Nagaraju. 2006. Molecular phylogeny of silkmoths reveals the origin of domesticated silkmoth, *Bombyx mori* from Chinese *Bombyx mandarina* and paternal inheritance of *Antheraea proylei* mitochondrial DNA. Mol. Phylogenet. Evol. 40: 419–427. 16644243 10.1016/j.ympev.2006.02.023

[CIT0002] Astaurov, B. L., and I. S. Rovinskaya. 1959. Chromosome complex of Ussuri geographical race of *Bombyx mandarina* M. with special reference to the problem of the origin of the domesticated silkworm, *Bombyx mori*. Cytology. 1: 327–332.

[CIT0003] Bai, X., G. Z. Zhang, M. M. Huang, S. P. Wei, L. Li, and S. T. He. 2015. Discussion on construction of Guangxi’s special silk culture by means of minority silk culture. China Sericult. 36: 77–81. [in Chinese]

[CIT0004] Bernt, M., A. Donath, F. Jühling, F. Externbrink, C. Florentz, G. Fritzsch, J. Pütz, M. Middendorf, and P. F. Stadler. 2013. MITOS: improved denovo metazoan mitochondrial genome annotation. Mol. Phylogenet. Evol. 69: 313–319. 22982435 10.1016/j.ympev.2012.08.023

[CIT0005] Cameron, S. L . 2014. Insect mitochondrial genomics: implications for evolution and phylogeny. Ann. Rev. Entomol. 59: 95–117. 24160435 10.1146/annurev-ento-011613-162007

[CIT0006] Chen, D. B., R. S. Zhang, H. X. Bian, Q. Li, R. X. Xia, Y. P. Li, Y. Q. Liu, and C. Lu. 2019. Comparative mitochondrial genomes provide new insights into the true wild progenitor and origin of domestic silkworm *Bombyx mori*. Int. J. Biol. Macromol. 131: 176–183. 30836184 10.1016/j.ijbiomac.2019.03.002

[CIT0007] Goldsmith, M. R., T. Shimada, and H. Abe. 2005. The genetics and genomics of the silkworm, *Bombyx mori*. Ann. Rev. Entomol 50: 71–100. 15355234 10.1146/annurev.ento.50.071803.130456

[CIT0008] Guan, Z. Y., G. Mo, D. N. Qin, C. M. Li, Z. Han, and P. Wu. 2014. Silkworm culture, preservation and development of Bai Ku Yao in Nandan. J. Guangxi Agric. 29: 77–80. [in Chinese]

[CIT0009] Huelsenbeck, J. P., and F. Ronquist. 2001. MRBAYES: Bayesian inference of phylogenetic trees. Bioinformatics. 17: 754–755. 11524383 10.1093/bioinformatics/17.8.754

[CIT0011] Jiang, Y. L . 1982. Origin and differentiation of domestic silkworm. Jiangsu Scientific and Technical Press, Nanjing. pp. 9–12. [in Chinese]

[CIT0012] Kong, W., and J. Yang. 2015. The complete mitochondrial genome of *Rondotia menciana* (Lepidoptera: Bombycidae). J. Insect Sci. 15: 48. 25888706 10.1093/jisesa/iev032 PMC4535477

[CIT0013] Kumar, S., G. Stecher, M. Li, K. Christina, and T. Koichiro. 2018. MEGA X: molecular evolutionary genetics analysis across computing platforms. Mol. Biol. Evol. 35: 1547–1549. 29722887 10.1093/molbev/msy096 PMC5967553

[CIT0501] Letunic, I., and P. Bork. 2021. Interactive Tree Of Life (iTOL) v5: an online tool for phylogenetic tree display and annotation. Nucleic Acids Res. 49: W293–W296. 33885785 10.1093/nar/gkab301 PMC8265157

[CIT0014] Li, B., C. Lu, Z. Y. Zhou, and Z. H. Xiang. 2000. The application of molecular quantitative genetics in silkworm breeding. Acta Sericologica Sin. 26: 25–28. [in Chinese]

[CIT0015] Li, A., Q. Zhao, S. Tang, Z. F. Zhang, S. Y. Pan, and G. F. Shen. 2005. Molecular phylogeny of the domesticated silkworm, *Bombyx mori*, based on the sequences of mitochondrial cytochrome b genes. J. Genet. 84: 137–142. 16131713 10.1007/BF02715839

[CIT0016] Li, R., Y. Li, K. Kristiansen, and J. Wang. 2008. SOAP: short oligonucleotide alignment program. Bioinformatics. 24: 713–714. 18227114 10.1093/bioinformatics/btn025

[CIT0017] Li, R., Y. Li, X. Fang, H. Yang, J. Wang, K. Kristiansen, and J. Wang. 2009. SNP detection for massively parallel whole-genome resequencing. Genome Res. 19: 1124–1132. 19420381 10.1101/gr.088013.108 PMC2694485

[CIT0018] Li, D., Y. Guo, H. Shao, L. C. Tellier, J. Wang, Z. H. Xiang, and Q. Y. Xia. 2010. Genetic diversity, molecular phylogeny and selection evidence of the silkworm mitochondria implicated by complete resequencing of 41 genomes. BMC Evol. Biol. 10: 81. 20334646 10.1186/1471-2148-10-81 PMC2856562

[CIT0019] Lu, C., S. H. Yu, and Z. H. Xiang. 2002. Molecular systematic studies on Chinese mandarina silkworm (*Bombyx mandarina* M.) and domestic silkworm (*Bombyx mori* L.). Agric. Sci. in China. 35: 94–101. [in Chinese]

[CIT0020] Lu, W. J., M. M. Lu, C. J. Yu, and Q. M. Qin. 2013. Enlightenment of Bai Ku Yao silkworm and silk production on modern silkworm breeding. Guangdong Canye. 47: 5–7. [in Chinese]

[CIT0021] Luo, H. C . 1993. The history of Guangxi’s sericulture. Guangxi Nationalities Publishing House, Nanning. pp. 5. [in Chinese]

[CIT0500] Nguyen, L. T., H. A. Schmidt, A. von Haeseler, and B. Q. Minh. 2015. IQ-TREE: a fast and effective stochastic algorithm for estimating maximum-likelihood phylogenies. Mol. Biol. Evol. 32: 268–274. 25371430 10.1093/molbev/msu300 PMC4271533

[CIT0022] Pan, M. H., Q. Y. Yu, Y. L. Xia, F. Y. Dai, Y. Q. Liu, C. Lu, Z. Zhang, and Z. H. Xiang. 2008. Characterization of mitochondrial genome of Chinese wild mulberry silkworm, *Bombyx mandarina* (Lepidoptera: Bombycidae). Sci. China Ser. C Life Sci. 51: 693–701. 18677597 10.1007/s11427-008-0097-6

[CIT0023] Rozas, J., A. Ferrer-Mata, J. C. Sánchez-Delbarrio, S. Guirao-Rico, P. Librado, S. E. Ramos-Onsins, and A. Sánchez-Gracia. 2017. DnaSP: DNA sequence polymorphism analysis of large datasets. Mol. Biol. Evol. 634: 3299–3302. 10.1093/molbev/msx248 29029172

[CIT0024] Sievers, F., A. Wilm, D. G. Dineen, T. J. Gibson, K. Karplus, W. Z. Li, R. Lopez, H. McWilliam, M. Remmert, and J. Söding. et al. 2011. Fast, scalable generation of high-quality protein multiple sequence alignments using Clustal Omega. Mol. Syst. Biol. 7: 539. 21988835 10.1038/msb.2011.75 PMC3261699

[CIT0025] Tan, L. and L. S. Jiang. 2012. Education values of silk culture in Baiku Yao ethnic group. Contemp. Educ. Cult. 4: 24–30. [in Chinese]

[CIT0026] Wen, Y. T . 2006. The meaning and the symbol of the dress decoration of Bai Ku Yao. J. Hechi Univ. 26: 112–115, 118. [in Chinese]

[CIT0027] Xia, Q., Y. Guo, Z. Zhang, D. Li, Z. L. Xuan, and Z. Li, et al. 2009. Complete resequencing of 40 genomes reveals domestication events and genes in silkworm (*Bombyx*). Science. 326: 433–436. 19713493 10.1126/science.1176620 PMC3951477

[CIT0028] Xiang, Z. H., J. T. Huang, J. G. Xia, and C. Lu. 2005. Biology of sericulture. China Forestry Publishing House, Beijing. pp. 1–5. [in Chinese]

[CIT0029] Xiang, H., X. Liu, M. Li, Y. Zhu, L. Wang, Y. Cui, L. Liu, G. Fang, H. Qian, and A. Xu. et al. 2018. The evolutionary road from wild moth to domestic silkworm. Nat. Ecol. Evol. 2: 1268–1279. 29967484 10.1038/s41559-018-0593-4

[CIT0030] Yan, T. T., R. X. Yin, Q. Li, P. Huang, X. N. Zeng, K. K. Huang, D. F. Wu, and L. H. H. Aung. 2012. Association of MYLIP rs3757354 SNP and several environmental factors with serum lipid levels in the Guangxi Bai Ku Yao and Han populations. Lipids Health Dis. 11: 141. 23107276 10.1186/1476-511X-11-141 PMC3496621

[CIT0031] Yukuhiro, K., H. Sezutsu, M. Itoh, K. Shimizu, and Y. Banno. 2002. Significant levels of sequence divergence and gene rearrangements have occurred between the mitochondrial genomes of the wild mulberry silkmoth, *Bombyx mandarina*, and its close relative, the domesticated silkmoth, *Bombyx mori*. Mol. Biol. Evol. 19: 1385–1389. 12140251 10.1093/oxfordjournals.molbev.a004200

[CIT0032] Zhang, G. Z., P. Wu, C. Lu, B. Y. Wei, W. G. Huang, Y. L. Zhang, H. M. Su, and L. H. Bi. 2017. A preliminary investigation of the basic economic traits of Yao silkworm, a specific silkworm strain in Guangxi. Guangxi Sericul. 54: 49–53. [in Chinese]

